# Oral and Intraperitoneal Administration of Quercetin Decreased Lymphocyte DNA Damage and Plasma Lipid Peroxidation Induced by TSA In Vivo

**DOI:** 10.1155/2014/580626

**Published:** 2014-04-23

**Authors:** Shu-Ting Chan, Yi-Chin Lin, Cheng-Hung Chuang, Rong-Jen Shiau, Jiunn-Wang Liao, Shu-Lan Yeh

**Affiliations:** ^1^Department of Nutritional Science, Chung Shan Medical University, No. 110 Section Jianguo North Road, Taichung 40201, Taiwan; ^2^Department of Nutrition, Hungkuang University, No. 34 Chung Chie Road, Shalu, Taichung County 43302, Taiwan; ^3^Department of Beauty Science, Chienkuo Technology University, Changhua 50094, Taiwan; ^4^Graduate Institute of Veterinary Pathology, College of Veterinary Medicine, National Chung Hsing University, Taichung 40227, Taiwan; ^5^Department of Nutrition, Chung Shan Medical University Hospital, Taichung 40201, Taiwan

## Abstract

Our previous study showed that quercetin enhances the anticancer effect of trichostatin A (TSA) in xenograft mice given quercetin intraperitoneally (10 mg/kg, 3 times/week). Herein, we investigate whether quercetin administered orally exerts such an effect and prevents the cytotoxic side effects of TSA. We found that quercetin given orally (20 and 100 mg/kg, 3 times/week) failed to enhance the antitumor effect of TSA although it increased the total quercetin concentration more than quercetin administered intraperitoneally in the plasma. The compound quercetin-3-glucuronide (Q3G) increased the most. However, quercetin administered intraperitoneally increased the total quercetin level in tumor tissues more than oral quercetin. Oral and intraperitoneal administration of quercetin similarly decreased lymphocyte DNA damage and plasma lipid peroxidation level induced by TSA. Furthermore, we found that the enhancing effect of Q3G on the antitumor effect of TSA and the incorporation of Q3G was less than that of quercetin in A549 cells. However, we found that A549 cells possessed the ability to convert Q3G to quercetin. In conclusion, different from quercetin administered intraperitoneally, quercetin administered orally failed to enhance the antitumor effect of TSA because of its metabolic conversion. However, it prevented TSA-induced DNA damage and lipid peroxidation.

## 1. Introduction

Quercetin is a common flavonoid found in various vegetal foods, such as onions, apples, and green leafy vegetables, and studies suggest that quercetin possesses various physiological properties including antioxidative and anti-inflammatory properties [1, 2]. For example, oral quercetin effectively decreases carbon tetrachloride-induced oxidative liver injury in mice and suppresses the depletion of glutathione peroxidase and superoxide dismutase [1]. Bureau et al. [2] demonstrated that quercetin reduces the inflammation-induced apoptotic neuronal cell death in a cell culture system. Our recent study showed that quercetin inhibits benzo[a]pyrene-induced lung inflammation in gerbils and in A549 cells which may be due to the downregulation of the JNK pathway [3]. In addition, growth evidence shows that quercetin may regulate intracellular signaling pathways, which are associated with cell proliferation and apoptosis, and then prevent cancer development [4, 5]. Quercetin may also enhance the effects of anticancer drugs [6, 7]. For example, quercetin significantly increases the anticancer effect of doxorubicin in breast cancer cells through several mechanisms and reduces the cytotoxic side effects of doxorubicin in nontumor cells [7]. Chen and Kang [8] found that quercetin in combination with trichostatin A (TSA), a histone deacetylase inhibitor with antiproliferation effects on various cancer cells [9, 10], cooperatively induces cell death in human leukemia HL-60 cells. Our previous study demonstrated that quercetin synergistically enhances the antitumor effect of TSA on human lung carcinoma cells (A549) through upregulation of p53 protein, at least in part [11]. We also found that quercetin administered by intraperitoneal (i.p.) injection decreases tumor size and upregulates the expression of p53 in tumor in xenograft mice, indicating that activating p53 may play an important role in the effect of i.p. injection quercetin [11]. However, whether oral administration of quercetin enhances the antitumor effect of TSA is unclear.

It has been shown that after quercetin intake, conjugated metabolites, such as quercetin glucuronides and quercetin sulfates, rather than quercetin aglycone are prevalent in human plasma due to its efficient phase II metabolism [12]. Similar results have been observed in animal studies [13, 14]. The biochemical and biophysical properties among quercetin and quercetin metabolites may be different because of structure modification [15], although some metabolites remain physiologically active [16, 17]. In addition, the incorporation efficiency of quercetin and its metabolites into cells may be different because of the different polarity of each compound. Thus, it is reasonable to assume that the influence of quercetin administered orally or intraperitoneally is different on the antitumor effect of TSA or other anticancer drugs.

The aim of this study was to investigate the effect of oral administration of quercetin on the antitumor effect and toxic effect of TSA in tumor bearing mice, compared with intraperitoneal administration. We also determined the distribution of quercetin and its metabolites in the plasma and tumor tissues to investigate the mechanisms that contributed to the effects of oral administration of quercetin. The doses of quercetin administered orally were at 20 and 100 mg/kg body weight 3 times/week, which were tenfold higher than the intraperitoneal doses. Besides, in our previous study we found that the plasma, which was obtained from Mongolian gerbils 2 h after quercetin feeding by gavage at 100 mg/kg body weight/week, induced A549 cell growth arrest in vitro [14]. In addition, we also used A549 cells to compare the intracellular accumulation and the enhancing effect of quercetin and quercetin-3-glucuronide (Q3G) on the antiproliferation effect of TSA.

## 2. Materials and Methods

### 2.1. Reagents

All chemicals used were reagent grade or higher. Quercetin and TSA were purchased from Sigma Chemical Co. (St. Louis, MO, USA). RPMI medium 1640, fetal bovine serum, trypsin, penicillin, streptomycin, sodium pyruvate, and nonessential amino acids were purchased from GIBCO/BRL (Rockville, MD, USA). Q3G and quercetin-3′-sulfate (Q3′S) was synthesized and purified according to the methods described previously [18, 19]. Mass spectrometric analysis was performed with electrospray ionisation in the negative-ion mode and in the range of* m/z* 100–700. The purity of Q3G and Q3′S was checked by HPLC and was found to be about 95% and ≧99%, respectively. The level of free quercetin in each of the synthetic compounds was undetectable (the limit of detection ≦6 pmole/injection).

### 2.2. The Animal Study

All study protocols were approved by the Institutional Animal Care and Use Committee at Chung Shan Medical University, and animal care followed the guidelines of the National Research Council [20]. Male nude mice aged 5 to 6 weeks were obtained from the National Laboratory Animal Center (Taipei, Taiwan). The animals were housed in specific pathogen-free conditions with an alternating 12-hour light:dark cycle. After being acclimated for 1 week, the animals were subcutaneously injected in the right flank with A549 cells at a dose of 5 × 10^6^ cells (in 200 *μ*L of matrigel; BD Biosciences, Franklin Lakes, NJ, USA). Tumor nodule volumes were measured once a week with the following formula: (*L*1 × *L*2^2^)/2 [21], where *L*1 is the long axis and *L*2 is the short axis of the tumor. Three weeks after cell injection, tumor nodules were palpable. The mean tumor volume was not significantly different among the groups at this time. The animals were then randomly assigned to the following six groups (*n* = 6/group) for 16 weeks: control, TSA, OL+TSA, OH+TSA, IL+TSA, and IH+TSA for TSA alone or in combination with quercetin treatment. TSA was given twice a week (0.5 mg/kg body weight) by i.p. injection, while quercetin was given 3 times a week by oral gavage (OL and OH, 20 and 100 mg/kg body weight, resp.) or by i.p. injection (IL and IH, 2 and 10 mg/kg body weight, resp.). Stock solutions of ethanol-TSA and ethanol-quercetin were freshly prepared before each injection and were diluted with 0.9% saline solution. The injection or gavage volume was 200 *μ*L (containing 25 *μ*L of stock solution and 175 *μ*L 0.9% saline solution) each time. The control group was given 200 *μ*L of vehicle (25 *μ*L ethanol and 175 *μ*L 0.9% saline solution) orally and intraperitoneally, respectively. All animals were allowed free access to a standard rodent diet (Lab 5001, Purina Mills, St. Louis, MO) and water during the study. During the 18-week experimental period, the body weights of the mice were recorded weekly. Blood samples were collected at weeks 4–10 (for determining quercetin concentration) and at week 17 (for determining lipid peroxidation level) from the retro-orbital plexus of the nude mice under deep isoflurane anesthesia. After the experiment, the animals were sacrificed. Blood and tissue samples were collected and were stored at −80°C until analyzed.

### 2.3. Measurement of Total Quercetin and Its Metabolites

We measured total or individual concentrations of quercetin and its metabolites, Q3G and Q3′S, in plasma and tumor tissues according to previously described methods [13, 18]. To determine the total concentration of quercetin, plasma (100 *μ*L) or homogenized tissue samples (0.03 g tissue in 300 *μ*L PBS) were incubated with a 5 *μ*L* Helix pomatia* enzyme mixture (~7500 U *β*-glucuronidase and ~750 U sulfatase in 0.5 M sodium acetate with 28 mM ascorbic aicd, pH 5.0) at 37°C for 2 h. To determine the individual concentration of quercetin metabolites, samples were added to the same volume of sodium acetate buffer but without hydrolyzed enzyme. Then 200 *μ*L of acetonitrile and 100 *μ*L of 20% H_3_PO_4_ were added to the mixture to deproteinize. After centrifugation at 2300 ×g and 10°C for 10 min, the supernatant was filtered and analyzed by HPLC with spectrophotometric detection at 370 nm. For HPLC analysis, an ODS3 reverse-phase silica column (250 × 4.6 mm, Phenomenex, Macclesfield, UK) was used. Solvents A (water : tetrahydrofuran : trifluoroacetic acid, 98 : 2 : 0.1,* v* :* v* :* v*) and B (acetonitrile) were employed to run a gradient program at a flow rate of 1.0 mL/min. The gradient program was as follows: 17% B (2 min), increasing to 25% B (5 min), 35% B (8 min), 50% B (5 min), and then to 100% B (5 min). To clean up the column, the solvent was maintained at 100% B for 5 min and then was returned to 17% B for the following 15 min.

### 2.4. Lipid Peroxidation and DNA Damage Assay

To determine the oxidative stress in the mice, we measured lipid peroxidation levels by determining thiobarbituric acid-reactive substances (TBARS) in the plasma using the fluorimetrical method which has been described previously [22]. The lymphocytes in the plasma, which were collected when the animals were sacrificed, were separated using Ficoll-Paque (GE Healthcare, Uppsala, Sweden). The DNA damage in the lymphocytes was determined by comet assay [23]. In brief, the lymphocytes were suspended in low-melting-point agarose in PBS at 37°C and were then placed on a frosted glass microscope slide precoated with a layer of 1% normal-melting-point agarose. After the application of a third layer of 1% normal-melting-point agarose, the slides were immersed in cold-lysing solution (10 mM Tris, 2.5 M NaCl, 100 mM Na_2_EDTA, 1% sodium N-laurylsarcosine, 1% Triton X-100, and 10% dimethylsulphoxide) for 1 h at 4°C. The slides were then placed in an electrophoresis tank, and the DNA was allowed to unwind for 15 min in the alkaline solution. Electrophoresis was performed using the method described by Collins et al. [24]. The images were analyzed by the Interactive Image Analysis Comet Assay III (Perceptive Instruments, Haverhill, UK), and DNA strand breaks were expressed as a percentage of DNA in the tail (% DNA in tail).

### 2.5. The Cell Culture

A549 cells were obtained from the American Type Culture Collection (Rockville, MD, USA). The cells were cultured in RPMI medium 1640 containing 10% (*v/v*) FBS, 0.37% (*w/v*) NaHCO_3_, penicillin (100 units/mL), and streptomycin (100 *μ*g/mL) at 37°C in a humidified incubator under 5% CO_2_ and 95% air. An equal number (2.5 × 10^4^/mL) of cells were incubated for 24 h before the various treatments. After being washed twice with PBS (pH 7.4, containing 137 mM NaCl, 2.7 mM KCl, 1.5 mM KH_2_PO_4_, and 8.1 mM Na_2_HPO_4_), the cells were incubated in fresh culture medium containing TSA (25 ng/mL equal to 82.5 nM) alone or in combination with quercetin, and Q3G (5 *μ*M) for indicated time. Stock solutions of ethanol-TSA (100 *μ*g/mL) were freshly prepared before each experiment. Quercetin and its metabolites were prepared in methanol at a stock concentration of 20 mM. The final solvent concentration in the medium was ≦0.05%. The medium was replaced every day. Cell growth was mainly measured by MTT colorimetric assay. The concentration of quercetin and Q3G incorporated into the cells was determined by the method mentioned above. In addition, according to the method described by Kawai et al. [25], we determined the intracellular *β*-glucuronidase activity by a colorimetric assay with phenolphthalein mono-*β*-glucuronide as the substrate. After 30 min of incubation of the cell lysate with a substrate, the formation of phenolphthalein aglycone was determined by monitoring the absorbance of samples at 550 nm.

### 2.6. Statistical Analysis

Values are expressed as means ± SD. We used one-way factorial analysis of variance followed by Duncan's multiple range test for comparisons of group means or Student's *t*-test for two-group comparisons. Differences were considered statistically significant at *P* < 0.05.

## 3. Results

### 3.1. Tumor Volume in Tumor Bearing Mice

**Figure 1:**
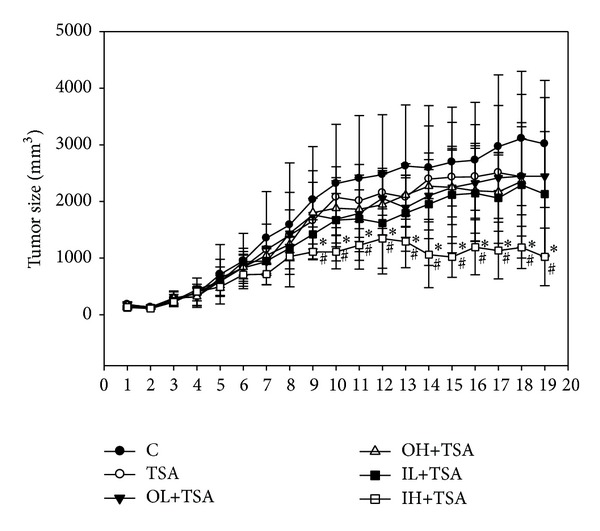
Effects of trichostatin A (TSA) alone or in combination with quercetin on tumor growth in tumor-bearing mice. Thirty-six male nude mice were injected with A549 cells and after 3 weeks were treated with TSA alone (0.5 mg/kg body weight) or in combination with quercetin (OL and OH, quercetin administered orally at 20 and 100 mg/kg body weight, resp.; IL and IH, quercetin administered intraperitoneally at 2 and 10 mg/kg body weight, resp.) for 16 weeks as described in Section 2. The control group was administered the vehicle only. Values (means ± SD, *n* = 6) with a ∗ denote a significant different from the control group, while a # is significantly different from the TSA group (*P* < 0.05).

Consistent with our previous study, TSA at 0.5 mg/kg body weight did not significantly decrease tumor size compared to the control group (Figure 1). OL+TSA, OH+TSA, and IL+TSA treatments also had no significant effect on tumor size in A549 tumor-bearing nude mice compared to the control group (*P* > 0.05). Only IH+TSA significantly decreased tumor growth in A549 tumor-bearing nude mice since week 9 compared with the control or TSA alone group. Quercetin alone, given orally and intraperitoneally, did not affect the tumor size; body weight of mice among groups was not significantly different (*P* > 0.05) (data not shown).

### 3.2. The Levels of Quercetin and Its Metabolites in Plasma and Tumor Tissues

**Figure 2:**
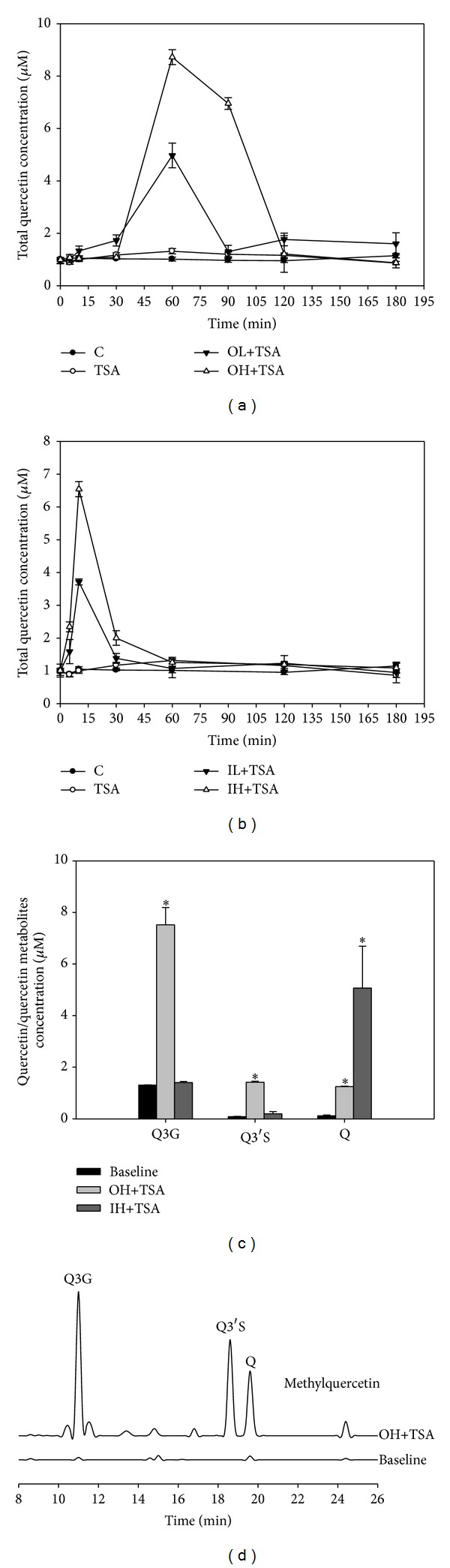
Total (a and b) and individual (c) concentrations of quercetin (Q) and its metabolites in plasma of tumor-bearing mice with various treatments as well as the HPLC chromatograms in the plasma (d). Thirty-six male nude mice were injected with A549 cells and after 3 weeks were treated with TSA alone (0.5 mg/kg body weight) or in combination with Q (OL and OH, Q administered orally at 20 and 100 mg/kg body weight, respectively; IL and IH, Q administered intraperitoneally at 2 and 10 mg/kg body weight, resp.) for 16 weeks as described in Section 2. The control group was administered the vehicle only. After various treatments for 4–10 weeks, total Q concentrations were determined after Q administration. Individual concentration of Q, quercetin-3-glucuronide (Q3G), and quercetin-3′-sulfate (Q3′S) in the plasma in OH+TSA and IH+TSA groups were determined at 1 h and 10 minutes after Q administration, respectively. Values are expressed as means ± SD (*n* = 6) and those of the same compound not sharing a common letter are significantly different (*P* < 0.05).

After treatment with TSA alone or in combination with quercetin for 4–10 weeks, the time course of the total quercetin concentration (the sum concentration of glucuronidated and sulfated quercetin and quercetin aglycone) after quercetin administration was determined by HPLC. The results showed that both oral and intraperitoneal administration of quercetin increased the total plasma concentration of quercetin in a dose dependent manner. The total quercetin level in the OL+TSA and OH+TSA groups reached a maximum approximately 1 h after quercetin administration (Figure 2(a)), while the maximum levels in the IL+TSA and IH+TSA groups appeared as early as 10 minutes (Figure 2(b)). We further determined individual levels of Q3G, Q3′S, and quercetin aglycone in the plasma at 1 h and at 10 minutes in the OH+TSA and IH+TSA groups, respectively. As shown in Figure 2(c), all Q3G, Q3′S, and quercetin levels in the OH+TSA groups increased significantly compared with those at the base line. However, Q3G was the major compound present in the plasma in the OH+TSA group and its level was about 5–6-fold of quercetin and Q3′S, meanwhile, in the IH+TSA group only the concentration of quercetin was significantly higher than that at the base line.  Figure 2(d) shows the HPLC chromatograms in the plasma of the OH+TSA group and at baseline. Methylated quercetin also increased in the OH+TSA group, but the concentration was markedly lower than Q3G and Q3′S (0.07±0.01 *μ*M).

**Figure 3:**
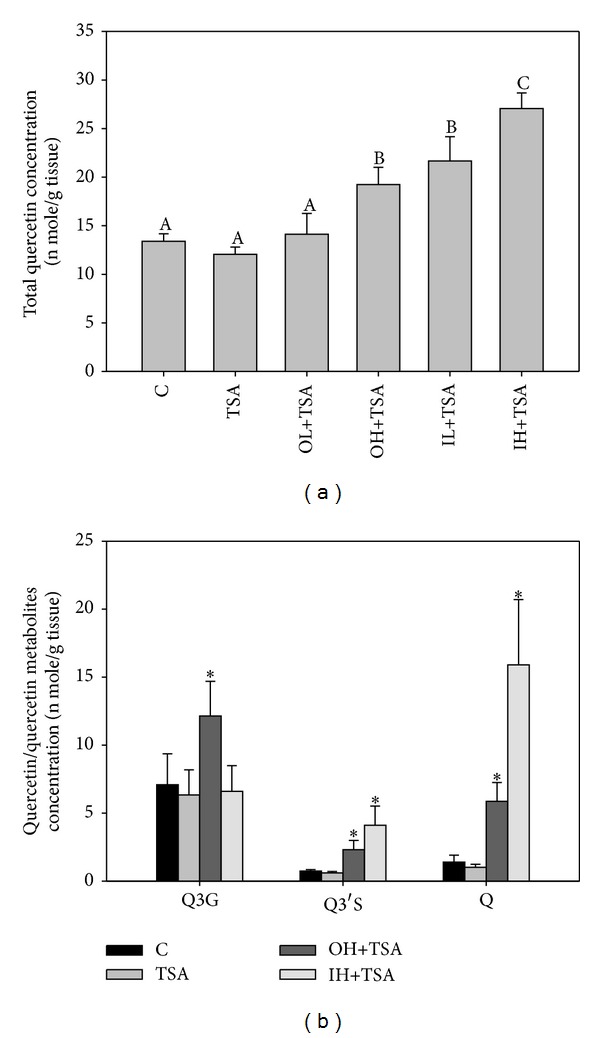
Total (a) and individual (b) concentrations of quercetin (Q) and its metabolites in tumor tissues of tumor-bearing mice with various treatments. Thirty-six male nude mice were injected with A549 cells and after 3 weeks were treated with TSA alone (0.5 mg/kg body weight) or in combination with Q (OL and OH, Q administered orally at 20 and 100 mg/kg body weight, respectively; IL and IH, Q administered intraperitoneally at 2 and 10 mg/kg body weight, resp.) for 16 weeks as described in Section 2. The control group was administered the vehicle only. After the mice were sacrificed, total and individual concentrations of Q, quercetin-3-glucuronide (Q3G), and quercetin-3′-sulfate (Q3′S) in the tumor tissues were determined. Values are expressed as means ± SD (*n* = 6) and those of the same compound not sharing a common letter are significantly different (*P* < 0.05).

After the animals were sacrificed, we determined the levels of quercetin and its metabolites in the tumor tissues. The results showed that the total quercetin level in the tumor tissues increased significantly in the OH+TSA, IL+TSA, and IH+TSA groups compared with the control group. The total quercetin levels were in the following order: IH+TSA > IL+TSA, OH+TSA > OL+TSA (Figure 3(a)). All Q3G, Q3′S, and quercetin levels increased significantly in the OH+T group compared with the control group (Figure 3(b)). On the other hand, the quercetin level increased significantly to about 11-fold in the IH+TSA group compared with the control group. In addition, in the IH+TSA group, the Q3′S level also increased significantly.

### 3.3. DNA Damage and Lipid Peroxidation

**Figure 4:**
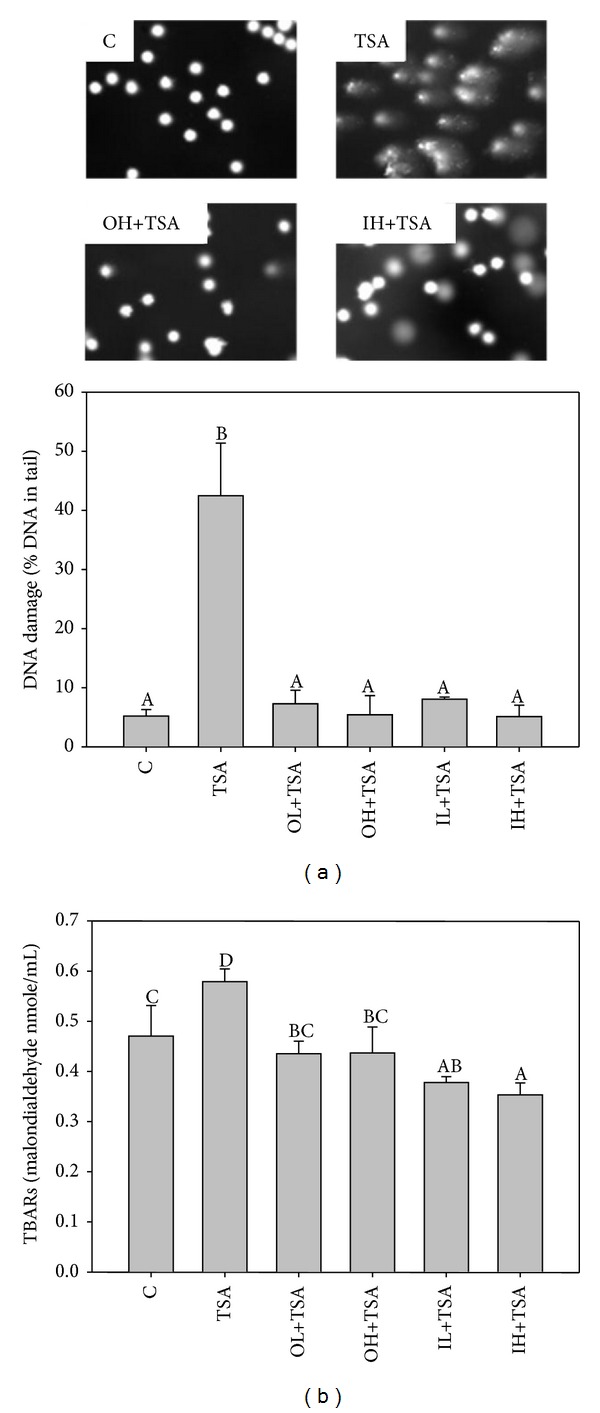
Lymphocyte DNA damage (a) and lipid peroxidation (TBARS level) (b) in tumor-bearing mice with various treatments. Thirty-six male nude mice were injected with A549 cells and after 3 weeks were treated with TSA alone (0.5 mg/kg body weight) or in combination with quercetin (OL and OH, quercetin administered orally at 20 and 100 mg/kg body weight, resp.; IL and IH, quercetin administered intraperitoneally at 2 and 10 mg/kg body weight, resp.) for 16 weeks as described in Section 2. The control group was administered the vehicle only. Values (means ± SD, *n* = 6) not sharing a common letter are significantly different (*P* < 0.05).

TSA alone significantly increased lymphocyte DNA damage up to 8.2-fold compared to the control group (Figure 4(a)). TSA also significantly increased the lipid oxidative product level in the plasma in tumor bearing mice (Figure 4(b)). In contrast, oral and intraperitoneal administration of quercetin significantly and similarly decreased TSA-induced lymphocyte DNA to the base line level (*P* < 0.05). In addition, quercetin administered orally and intraperitoneally decreased lipid peroxidation induced by TSA. The TBARS levels in the groups with quercetin administered intraperitoneally were even lower than in the control group (*P* < 0.05).

### 3.4. The Effect and the Incorporation of Q3G in A549 Cells Exposed to TSA

**Figure 5:**
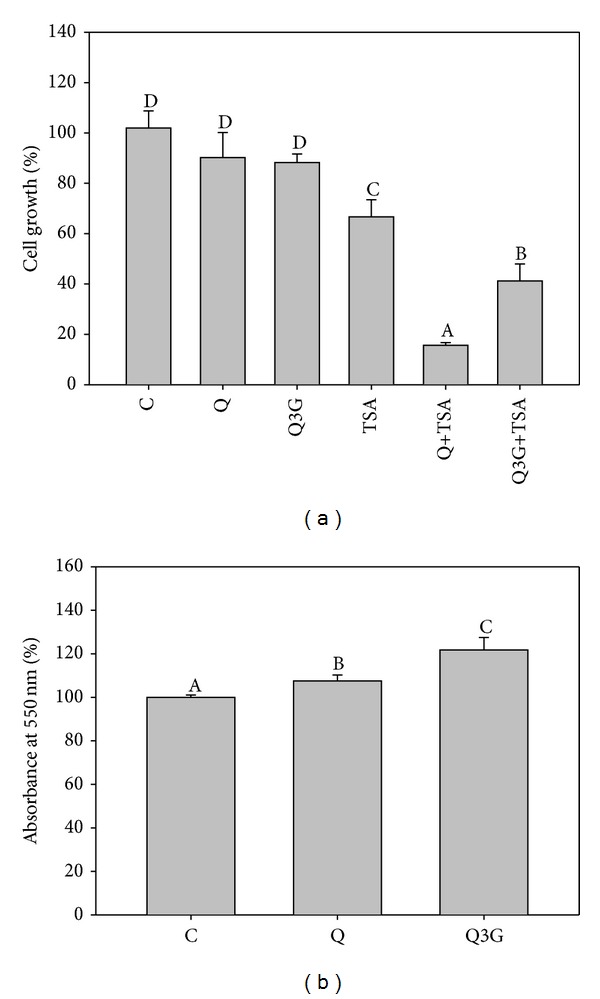
Effects quercetin (Q) or quercetin-3-glucuronide (Q3G) on the growth (a) or intracellular *β*-glucuronidase activity (b) of A549 cells exposed to trichostatin A (TSA) or not. To determine cell growth, the cells were incubated with TSA (25 ng/mL), 5 *μ*M Q, or Q3G alone or combined for 72 h. Meanwhile, the cells were incubated with 5 *μ*M Q or Q3G only for 1 or 4 h to study the intracellular *β*-glucuronidase activity which was assayed by determining the formation of phenolphthalein after incubation of cell lysates with phenolphthalein monoglucuronides. Values (means ± SD, *n* = 3) not sharing a common letter are significantly different (*P* < 0.05).

Because Q3G was the dominant metabolite present in the plasma after oral administration of quercetin, we compared the enhancing effect of Q3G and quercetin at 5 *μ*M (this concentration is comparable to the maximum level in the plasma after quercetin administration) on the antiproliferation effect of TSA (25 ng/mL) in A549 cells. TSA alone inhibited the growth of A549 cells by 35% after incubation for 72 h (Figure 5(a)). Both quercetin and Q3G significantly enhanced the antiproliferation effect of TSA. However, the enhancing effect of quercetin was significantly better than Q3G (76.7% versus 38.2%, *P* < 0.05). Furthermore, we compared the levels of quercetin and its metabolites in A549 cells incubated with quercetin or Q3G. After incubation with quercetin for 1 and 4 h, the intracellular quercetin concentration increased significantly in a time-dependent manner (Table 1). Q3G incubation also significantly increased the intracellular Q3G concentration in a time-dependent manner. However, the increased level of quercetin + Q3G induced by Q3G incubation was only 43% of that induced by quercetin incubation at 4 h. In addition, we surprisingly found that Q3G incubation for 4 h also increased the intracellular quercetin level. To investigate whether A549 cells possessed the ability to converted Q3G to quercetin, we determined intracellular *β*-glucuronidase activity. As shown in Figure 5(b), after incubation with Q3G for 4 h, *β*-glucuronidase activity increased significantly in A549 cells compared with the control and quercetin groups.

## 4. Discussion

Quercetin, an antioxidative flavonoid widely distributed in plants, has been shown to possess cancer preventive effects on various cancer cells. In addition, growing evidence shows that quercetin may enhance the effect of anticancer drugs [7, 11]. In our previous study [11] we found that quercetin administered intraperitoneally at 10 mg/kg body weight significantly enhanced the antitumor effect of TSA in tumor bearing mice which may be due to the upregulation of p53 expression. However, in the present study, the results demonstrated that quercetin (20 and 100 mg/kg body weight, 3 times per week) given by gavage failed to exert such an effect, despite the level of total quercetin (quercetin plus its metabolites) in the plasma being higher and the duration time being longer in the OH+TSA group compared to the IH+TSA group. The contributing factor for the ineffectiveness of oral quercetin administration could be associated with the metabolic conversion of quercetin in vivo. A lot of chemopreventive compounds including phytochemicals may have different biological effects between oral administration and intraperitoneal administration because the absorbance and metabolism of these compounds affect their concentrations and structures in vivo [26, 27]. For example, although curcumin, a phytochemical present in herbs and spices, possesses various biological activities and inhibits the growth of various cancer cells in vitro, its low systemic bioavailability following oral dosing has been suggested to limit access of sufficient concentrations for pharmacological effect in certain tissues [26]. However, in the gastrointestinal tract, curcumin may attain biologically active levels.

Consistent with previous studies [13, 14], the present study also showed that oral administration of quercetin mainly increased quercetin metabolites, especially Q3G, in the plasma of mice. In contrast, i.p. injections of quercetin mainly increased the level of quercetin itself. These data suggest that the overall enhancing effects of quercetin metabolites on the antitumor effect of TSA are less than quercetin itself in vivo. Our in vitro study supports this postulation because we found that Q3G had little effect on the antiproliferation effect of TSA in A549 cells than quercetin itself (Figure 5). We cannot rule out that the other quercetin metabolites, such as Q3′S and methylated quercetin, may be more efficient than Q3G. However, because Q3G is the dominant product found in plasma in nude mice (the concentrations of Q3G, Q3′S, and methylated quercetin: 7.5 *μ*M, 1.4 *μ*M, and 0.07 *μ*M, resp.), we speculated that the effects of quercetin metabolites are mainly attributed to the effect of Q3G. Growing evidence shows that the biochemical and biophysical properties among quercetin and quercetin metabolites are different [4]. However, few studies address the effect of Q3G on cancer development. A recent study using doses of 21–210 *μ*M, which, however, were 4–40-fold of those used in our study, found that Q3G suppresses MCF-7 breast cancer cell migration induced by plasmin [28].

Furthermore, we determined the levels of quercetin and its metabolites in tumors. To our knowledge, this is the first report showing the presence of quercetin and its metabolites in tumor tissue. In contrast to the findings in plasma samples, the level of total quercetin in tumor tissues in the IH+TSA group was higher than in the OH+TSA group. Quercetin was still the dominant compound present in tumor tissues in the IH+TSA group while Q3G was the major compound present in tumor tissues in the OH+TSA group. These findings suggest that quercetin entered cells more efficiently than its metabolites which may be due to the higher polarity of Q3G and Q3′S than that of quercetin. Our in vitro study also supports the in vivo finding; that is, after incubation with Q3G, the total level of quercetin in cells was lower than that of quercetin incubation (Table 1).

An unexpected finding herein was that oral administration of quercetin and Q3G exposure increased quercetin levels in tumor tissues and A549 cells, respectively, suggesting that A549 cells may produce glucuronidase and deconjugated Q3G into quercetin. It has been suggested that quercetin metabolites may convert to quercetin due to some physiological conditions [25, 29]. Kawai et al. [25] demonstrated that Q3G was selectively incorporated into activated macrophages and then deconjugated to quercetin. Our further study with cell culture experiments demonstrated that Q3G incubation in A549 cells significantly induced intracellular *β*-glucuronidase activity, indicating the possibility that Q3G converts to quercetin and increases its bioactivity in lung tumor cells. However, more studies are warranted to investigate whether other lung cancer cells possess such a feature.

TSA has been shown to induce oxidative stress and leads to apoptosis of HeLa cervical cancer cells [30]. Our previous study showed that TSA increases oxidative stress and DNA damage in A549 cells [31]. In the in vivo study, we found that TSA also increased lipid peroxidation and lymphocyte DNA damage in tumor-bearing mice. In fact, various chemotherapy drugs exert toxic effects on normal cells which lower the treatment tolerance of patients. Although quercetin administered orally at 20 and 100 mg/kg body weight failed to enhance the antitumor effect of TSA, the present study demonstrated that oral supplementation with quercetin decreased the TSA-induced toxic effects without influencing its antitumor effect. The protective effects of quercetin given orally were comparable to that given intraperitoneally. A recent study also demonstrated that quercetin (50 mg/kg/day, intraperitoneally) reduces cisplatin nephrotoxicity in rats without compromising its antitumor activity [32]. The study showed that the underlying mechanisms are associated with the antioxidative and anti-inflammatory activity of quercetin. Although the precise mechanisms under the protective effect of oral administration of quercetin remain unclear in our study, these results suggest that oral intake of quercetin has a potential protective compound against chemotherapy toxicity without compromising the efficiency of chemotherapy.

In conclusion, the present study showed that the oral administration of quercetin at doses of 20 and 100 mg/kg body weight, 3 times per week, failed to enhance the antitumor effect of TSA. This result was associated with the metabolic conversion of quercetin in vivo. However, quercetin administered orally diminished TSA-induced adverse effects in nude mice and the effect was similar to that of i.p. injections. Further studies are warranted to investigate the effect of quercetin administered orally at higher doses.

## Figures and Tables

**Table 1:** Accumulation of quercetin (Q) and quercetin-3-glucuronide (Q3G) in A549 cells^1^.

Group	Compound	
	Q3G (*n* mole/g protein)	Q (*n* mole/g protein)
	1 h	
C	1.39 ± 0.01^a^	0.70 ± 0.35^a^
Q	1.40 ± 0.01^a^	2.36 ± 0.69^b^
Q3G	1.99 ± 0.03^b^	0.77 ± 0.21^a^

	4 h	
C	1.39 ± 0.01^a^	0.70 ± 0.35^a^
Q	1.88 ± 0.09^b^	10.70 ± 1.14^c^
Q3G	2.48 ± 0.01^c^	2.97 ± 0.10^b^

^1^The cells were incubated with 5 *μ*M quercetin Q or Q3G for 1 and 4 h. Values (means ± SD, *n* = 3) of the same compound and the same time point not sharing a common letter are significantly different (*P* < 0.05).
